# Supplementary material for: Patterns of Intron Gain and Loss in Fungi
Source: PLoS Biol. 2004 Nov 30;2(12):e422. doi: 10.1371/journal.pbio.0020422 (PMC532390; doi:10.1371/journal.pbio.0020422)
Supplement: Table S1 — Also available at http://genes.mit.edu/NielsenEtAl/. (4.3 MB ZIP). [file pbio.0020422.st001.zip › NielsenEtAl/html/865.html]

AN3901.1.NCU08272.1.MG01723.1.FG05328.1


```
 CLUSTAL W (1.82) Multiple Sequence Alignments - Introns Inserted


Sequence 1: MG01723.1	494 aa
Sequence 2: FG05328.1	502 aa
Sequence 3: NCU08272.1	501 aa
Sequence 4: AN3901.1	493 aa
Alignment Length: 509 aa
Number Identitical Residues: 309 aa
Alignment Score (without introns) 14135


MG01723.1 	----MPLTGAEVAKHDSSQSCWVIVH~GKAYDVTEFLPE~HPGGAKIILKYA~GKDATEE
NCU08272.1	MAPQTPLTGVEVAKHNTQDDCWVIVH~GKAYDITEFLPE~HPGGMKIILKYA~GKDATEE
FG05328.1 	----MVLKGGEVAEHNSADSCWVIVH0GKAYDVTEFLPE1HPGGKKIILKYA0GKDATEA
AN3901.1  	-MTNNKLTGADVAKHNSKDSCWVIVH~GKAYDVTEFLPE1HPGGQKIILKYA~GKDATEE
          	  ..  *.* :**:*:: :.****** *****:****** **** ******* ****** 

MG01723.1 	FDPIHPPDTLDKYLDKSKHMGPVDMSTVAHVQKAADPEEEARLRRVADIPLLEQCYNLLD
NCU08272.1	FDPIHPPDTLEKYLAKDKHLGPVDMSTVVTEKAEVDPEEQARLKRIEEMPLLEQCYNLLD
FG05328.1 	YEPIHPPDTLDKFLEASKHLGPVDMNTVQQETKEVDPEEEERLQRMEQKPLLSQCYNLFD
AN3901.1  	FDPIHPPDTLDKYLDSSKHLGEVDMATVEQEEKAHDPEEDARQERIERMPPLSACYNLLD
          	::********:*:*  .**:* *** **       ****: * .*:   * *. ****:*

MG01723.1 	FEAVARRVMKKTAWGYYSSAADDEI0TFRENHSAFHRIWFRPKVLVDVENVDVSTTMLGT
NCU08272.1	FEAVAKRVMKKNAWAYYSSAADDEI0TLRENHAAFHRIWFRPKVLVDVEKVDFSTTMLGT
FG05328.1 	FEAVARRVMSKVAWGYYSSAADDEI0TMRENHSAFHRIWFRPQILVDVENIDFSTTMLGT
AN3901.1  	FETVARSVMKKTAWAYYSSGADDEI0TMRENHQAFQKIWFRPRVLVDVENVDFSTKMLGT
          	**:**: **.* **.****.***** *:**** **::*****::*****::*.**.****

MG01723.1 	KTALPFYVTATALGKLGNPEGEVCLTKAAGKHNVIQMIPTLASCAFDEIMDAAVPGQVQW
NCU08272.1	KVDIPFYVTATALGKLGHVEGEVLLTRAAKKHNVVQMIPTLASCAFDEIMDAAEGDQVQW
FG05328.1 	KTDIPVYVTATALGKLGNPEGEVVLTRAAAKHNVIQMIPTLASCSFDEIVDAKAGDQVQW
AN3901.1  	KCSIPFYVTATALGKLGNPEGEVVLTRAAHDHDVIQMIPTLASCSFDEIVDARRGDQVQW
          	*  :*.***********: **** **:** .*:*:*********:****:**   .****

MG01723.1 	LQLYVNKDREVTKRIVQYAEKRGCKGLFITVDAPQLGRREKDMRSKFEDPGTSVQQ--GQ
NCU08272.1	LQLYVNKDRAITERIIKHAEKRGCKALFITVDAPQLGRREKDMRVKFTDDGSNVQK--GH
FG05328.1 	LQLYVNKDRAITKKIVQHAEKRGCKGLFITVDAPQLGRREKDMRSKFTDPGSHVQE--GT
AN3901.1  	LQLYVNKDRAITKRIIEHAEARGCKGLFITVDAPQLGRREKDMRSKFSDVGSNVQATGGD
          	********* :*::*:::** ****.****************** ** * *: ** :.* 

MG01723.1 	TTDNSQGAARAISSFIDPALSWKDLPWFRSITKMPIVLKGVQRVEDVLKAVDAGMDGVIL
NCU08272.1	ETNRNEGAARAISSFIDPALSWKDIPWFQSVTKMPIILKGVQRVEDVIKAVEAGVQGVVL
FG05328.1 	DTDNSQGAARAISTFIDPALSWKDIAWFQSITSMPIILKGVQRVEDVLKAIDYGCQGVVL
AN3901.1  	EVDRSQGAARAISSFIDPSLSWKDIPWFQSVTKMPIVLKGVQCVEDVLRAVEAGVQGVVL
          	 .:..:*******:****:*****:.**:*:*.***:***** ****::*:: * :**:*

MG01723.1 	SNHGGRQLEFARSGIEILAETMPVLRSMGLQDKIEVYLDGGVRRGTDIIKALCLGAKGVG
NCU08272.1	SNHGGRQLEFARSGIEVLAETMPVLRELGLEDKIEVYIDGGIRRATDILKALCLGAKGVG
FG05328.1 	SNHGGRQLEFARSAIEVLAETMPILRERGLENKIEIFIDGGIRRGTDILKALCLGARGVG
AN3901.1  	SNHGGRQLDTAPSGIEVLAQVMPILRERGWENRIEIFIDGGIRRATDILKALCLGAKGVG
          	********: * *.**:**:.**:**. * :::**:::***:**.***:*******:***

MG01723.1 	IGRPFLYAMSAYGVQGVDRAMQLLKDELEMNMRLIGCTSIDQLSPSLVDTKSLTYHGN~G
NCU08272.1	IGRPFLYAMSAYGFDGVDRAMQLLKDEMEMNMRLIGATKIEDLNPGMLDLRSLYNHG-~A
FG05328.1 	IGRPFLYAMSTYGEAGVIRAMQLLKDELEMNMRLIGASKIEDLHPGMLDLRSLFQHG-~A
AN3901.1  	IGRPFLFAMSAYGQPGVNRAMQLLKDELEMNMRLIGAQKIADLNPSMVDVRGLVGGHI1P
          	******:***:**  ** *********:********. .* :* *.::* :.*       

MG01723.1 	TPIDNLSHSIYDPLANPPQRT--G-------AKL
NCU08272.1	PPTDTLSNISYDALISPAQRVKFAE-----KSKI
FG05328.1 	VPSDNLSSTVYDPLAVPAQRPKAGPEQGAPKAKL
AN3901.1  	LMFNWLSSAPDFNNAYINA---------------
          	   : **
```
